# Application of Green Technologies in Design-Based Extraction of *Celastrus paniculatus* (*Jyotishmati*) Seeds, SEM, GC-MS Analysis, and Evaluation for Memory Enhancing Potential

**DOI:** 10.3389/fnut.2022.871183

**Published:** 2022-05-18

**Authors:** Ashwani Arya, Deepak Kaushik, Rafa Almeer, Simona G. Bungau, Amany A. Sayed, Mohamed M. Abdel-Daim, Saurabh Bhatia, Vineet Mittal

**Affiliations:** ^1^Department of Pharmaceutical Sciences, Maharshi Dayanand University, Rohtak, India; ^2^Department of Zoology, College of Science, King Saud University, Riyadh, Saudi Arabia; ^3^Department of Pharmacy, Faculty of Medicine and Pharmacy, University of Oradea, Oradea, Romania; ^4^Zoology Department, Faculty of Science, Cairo University, Giza, Egypt; ^5^Pharmacology Department, Faculty of Veterinary Medicine, Suez Canal University, Ismailia, Egypt; ^6^Natural and Medical Sciences Research Center, University of Nizwa, Nizwa, Oman; ^7^School of Health Science, University of Petroleum and Energy Studies, Dehradun, India

**Keywords:** *Celastrus paniculatus*, antioxidant, anti-cholinesterase, green approach, Box-Behnken design, scanning electron microscopy, gas chromatography, response surface methodology

## Abstract

**Background:**

The *Celastrus paniculatus* (CP), commonly known as *Jyotishmati*, is considered as “elixir of life” by Indian people for the prevention or management of many ailments. The seed powder and its extract have widely used commercially for the preparation of various Ayurvedic formulations for the improvement of memory. CP seeds were generally extracted by conventional extraction methods (CEMs) which are assumed to impact environment burden and also produce low extract yield. Green extraction with possible improvement in extract yield has always been the need of hour for selected medicinal plant.

**Objective:**

In the present research, we aimed to optimize the different extraction factors in microwave and ultrasound-based extraction. The various extracts obtained in conventional and green methods are also evaluated for the possible improvement in memory enhancing potential.

**Materials and Methods:**

The selected medicinal herb was extracted by CEM (maceration and percolation). In green methods such microwave-assisted extraction (MAE) and ultrasound assisted-extraction (UAE), various parameters were optimized using Box-Behnken design coupled with response surface methodology. The scanning electron microscopy (SEM) and gas chromatography–mass spectroscopy (GC-MS) analyses were also done to confirm the possible improvement in concentration of plant actives. The Swiss albino mice were used to evaluate memory enhancing potential of different extracts.

**Results:**

At the optimized conditions MAE and UAE the extraction yield, total phenolic content (TPC) and Total flavonoid content (TFC) are significantly improved. The GC-MS analysis further confirms the improvement in concentration of certain fatty acid esters, pilocarpine, and steroidal compounds in optimized extracts. The optimized extracts also exhibited the significant improvement in behavioral parameters, oxidative stress-induced parameters, and acetylcholinesterase inhibitory potential.

**Discussion and Conclusion:**

From the results, we can say that the application of green technologies in design-based extraction of selected herb not only significantly reduces the extraction time but also improves the extract yield and concentration of plant actives. In nutshell, it can be concluded that the green approaches for extraction of seeds of *Celastrus paniculatus* could be scale up at a commercial level to meet the rising demand for herbal extract.

## Introduction

Extraction is the first step to extract or isolate the secondary metabolites from the botanicals. Nowadays, a serious concern has been shown by all the countries in saving the environment by reducing the carbon load. Even the industries have also realized that the future existence is only possible if we follow the “green approach” in extraction of herbs (1). The conventional extraction methods (CEMs) such as maceration and Soxhlet are very time-consuming processes that require relatively large quantities of organic solvents and produce low extract yield. The non-conventional methods such as microwave-assisted extraction (MAE), ultrasound-assisted extraction (UAE), pressurized liquid extraction (PLE), supercritical fluid extraction (SFE), and enzyme-assisted extraction (EAE) could be successfully employed for the extraction of herbs in place of CEMs. These novel methods of extraction not only proved to be energy efficient but also ensure the improved yield of extracts and phytoconstituents. Several researchers identified the significance of traditional herbs as an inexpensive basis to healthcare system for the prevention of many health ailments (2, 3). A diverse nature of secondary metabolites has evolved from the plants. These secondary metabolites are involved in various types of interactions inside the plant and also play a crucial role in the scientific community for the development of pharmaceutical formulations. Moreover, a huge amount of secondary metabolites from the herbs can also be used as the additives in food supplement and as flavor or colors in the pharmaceutical formulations (1, 2). Thus, the application of green methods in the extraction of herbs could further help in sustainable supply of extracts for industries in an economical manner.

*Celastrus paniculatus* (CP) Willd belonging to the *celeastraceae* family is a woody, large, climbing shrub of about 10–18 m height endangered plant that is widely and commonly available all over India. In traditional ayurvedic medicine, CP has been utilized for neuroprotective, memory enhancer, anxiolytic, antibacterial, antifungal, wound healing, and anti-inflammatory activities (4–6). Strong antioxidant potential of CP has also been established through radical scavenging activities (7). Several reports demonstrated that seeds of CP are used for brain disorders and to improve memory processes (8). From the ayurvedic system of medicine, the CP is reported for sharpening the memory and stimulating intellectual property. Several studies reported that the seed extract of CP enhances cognitive activity which is due to increased acetylcholine in the brain. Also, antioxidant property was proved to be responsible for its enhanced cognitive activity (9). Several phytochemical studies have revealed previously that extract from the seed of *CP* comprised of a complex mixture of components such as stearic, linoleic, palmatic, oleic, and linolenic acids, sesquiterpene alkaloids such as celapanigin, celapanin, and celapagin, phytosterols, and sesquiterpene polyol ester including campesterol, stigmasterol, and β-sitosterol. This herb is also reported as a novel candidate in the development of a therapeutic approach in the prevention of Alzheimer's disease (7, 10).

Nowadays, green technologies are frequently employed for the extraction of plants. MAE and UAE methods were proved to improve the extraction yield (EY) in less time with lower amount of solvent consumption (11, 12). Optimization of process could further help in the selection of exact extraction conditions which could be employed for the production of extract at a large scale (13). Hence, keeping in view about the pharmacological significance and industrial prospects of the selected herb, it is decided to optimize the different parameters for the extraction of *CP* by MAE and UAE techniques with the possible aim to improve the yield of extract and phytoconstituents in an energy-efficient manner. Moreover, the different extracts are also evaluated for *in vivo* antioxidant and anti-cholinesterase activity. The scanning electron microscopy (SEM) of drug samples was also carried out to evaluate the microstructural change after the different extraction methods. The gas chromatography–mass spectroscopy (GC-MS) analysis of different extracts was also done to confirm the alteration in concentration of phytoconstituents after application of green technologies in extraction of selected medicinal plant.

## Materials and Methods

### Plant Sample and Solvents

The seeds of *CP* Willd plant commonly known as “*Jyotishmati*” were purchased from a local market in Hisar and authenticated by Dr. Sunita Garg, Emeritus Scientist, CSIR-NISCAIR, New Delhi vide reference number NISCAIR/RHMD/Consult/2018/3157-06-1 dated 19/03/2018. The solvents and chemicals used in the study are of analytical grade.

### Conventional Extraction Methods

After the procurement of plant material (seeds), these were dried in shade and powdered. The coarse powder of plant sample was sieved (size 60) and kept in air-tight container before further processing for the extraction by different methods.

#### Extraction by Maceration

The powder sample of herb was weighed (10 g) and was kept in a conical flask along with the solvent (ethanol). After 8 days, the extracted sample mixture was filtered and concentrated at temperature not more than 60°C. The EY (w/w) was measured, and extract was kept in air-tight container till further analysis.

#### Extraction by Percolation

The powder of the herb (10 gm) was also extracted by continuous hot percolation method in Soxhlet apparatus using ethanol as menstruum at 60°C until complete exhaustion of the sample (about 9 h) (6, 7). The extract was filtered through Whatman filter paper, concentrated and the residue was weighed to calculate the yield (w/w) (6, 7).

### Microwave-Assisted Extraction

The experiments on MAE were carried out in close-type microwave synthesis apparatus (CEM Matthewis, NC, and USA). During the extraction process, temperature was kept constant at 80°C throughout the study. Powder sample (0.4 g) was extracted at different operating conditions of microwave power, time, concentration of solvent, and solvent to drug ratio (ml/g) as suggested by the software. The seed powder (0.4 gm) was mixed with the solvent in a quartz vessel and irradiated with microwave radiations for the specific time. After the extraction process, the solution was kept at room temperature for cooling, centrifuged at 5,000 rpm for 15 min, and then filtered. The extract was concentrated in a rotary evaporator and the weight of dry extract was measured for the calculation of percentage yield (w/w) (14).

### Ultrasound-Assisted Extraction

Ultrasound-assisted extraction of the selected herb was carried out using an Ultrasonic Processor (Model: UP-800, Chrome Tech. Co, Ltd, Taiwan). The ultrasonic processor is equipped with a sound abating enclosure, elevator, and temperature control sensor. For the extraction process, the powdered material (1 gm) was kept along with the solvent and the ultrasonic probe tip was immersed to produce the sound waves. Moreover, some parameters such as frequency (24 Khz), pulse (0.5 s), probe diameter (5/8”mm), and intensity (high) was kept fixed based on the some preliminary experiments. UAE of sample was carried out at the different experimental conditions as suggested by the design software. After extraction, the solution was centrifuged at 5,000 rpm for 15 min, filtered, and concentrated in a rotary evaporator (15).

### Experimental Design

The seed powder of selected plant has been used for extraction by MAE and UAE techniques. In this research, the various parameters such as microwave power, X_1_, (100–300 watt), extraction time, X_2_, (1–10 min), solvent concentration, X_3_, (20–80%), and solid/liquid ratio, X_4_, (5–50 ml/g) of MAE were optimized using the Box-Behnken design (BBD) coupled with response surface methodology (RSM). The total 29 experiments were carried out as suggested by the software. Similarly, the various factors of UAE such as sonication time, X_1_, (5–20 min), solvent concentration, X_2_, (50–90%), and volume of solvent, X_3_, (10–30 ml) were optimized using BBD. The total of 17 experiments run were performed as per the conditions obtained after applying the BBD. In both the techniques, the extraction parameters were optimized for the response like EY (16). Further, the design expert software (7.0.3, Statease Inc, Minneapolis, USA) was used to design, analyze the results, and to get the response surfaces.

### Total Phenolic Content

Folin–Ciocalteu method was utilized to evaluate the total phenolic content (TPC) of the different extracts. Plant extract (0.5 g) was dissolved in methanol (10 mg/ml), The Folin–Ciocalteu reagent (1.5 ml) was added in this solution. After 10 min, 1.5 ml of aqueous Na_2_CO_3_ solution (25%) was mixed. After stirring, the mixture was incubated for 30 min at 45°C on a water bath. The absorbance of the resultant sample mixture was measured at 760 nm with the aid of UV-visible spectrophotometer (UV-1800, Shimadzu Scientific Instruments Private Limited) against the blank. Gallic acid was used as a standard solution and the different concentrations (50–250 μg/ml) of it were used to generate the straight-line equation. The total phenolic content was determined and expressed as mg GAE/g of extract. The absorbance was measured in triplicate, and results were expressed as mean ± standard deviation (SD) (13).

### Total Flavonoid Content

Total flavonoid content (TFC) was determined by aluminum chloride method. The plant extract was dissolved in methanol in a concentration of 10 mg/ml. About 1.5 ml of the aluminum chloride solution (2%) was added in this mixture. For 1 h, the sample mixture was kept at room temperature and absorbance was measured against the blank sample at 415 nm in UV-visible spectrophotometer. The TFC was estimated as mg RUE/g of extract. The study was performed in a triplicate, and results were expressed as mean ± SD (13).

### Scanning Electron Microscopy

The powder of drug samples was examined by SEM for comparative microstructure analysis featured after and before extraction with various extraction techniques. For the preparation of samples, residues after exhaustion of extraction process were dried at 40–50°C under vacuum for at least 2 h, and samples were used after sputtered coated with gold for SEM analysis (17).

### Gas Chromatography–Mass Spectroscopy of Extracts

The various extracts of *CP* Willd obtained by different extraction methods (maceration, Soxhlet, MAE, and UAE) were analyzed for GC-MS using Shimadzu QP-2010 with Thermal Desorption System TD 20 equipped with MS capillary column. For GC-MS analysis, an electron ionization system was used having an energy of 70 eV. Helium (He) was used as the carrier gas and the temperature of injector (260°C) with a flow rate was set at 1.5 ml/min. The initial temperature of the column was set at 70°C for 2 min, and it was elevated to 150°C at a rate of 3°C/min which was held for another 10 min and finally increased to 250°C at the rate of 4°C per minute. The sample injection volume (1.0 μl) was manually injected in split mode. The chromatogram was recorded and components were identified by comparing the results with library of compounds (18).

### Evaluation of Memory Enhancing Potential

#### Animals

The research protocol was approved by the institutional animal ethical committee (IAEC), MDU, Rohtak, vide reference number1767/RE/S/14/CPCSEA/CAH/76-85 dated 26-02-2021. The Swiss albino mice (25–30 g) were purchased from the disease-free small animal house of Lala Lajpat Rai University of Veterinary and Animal Sciences (LLRU-VAS), Hisar, India. The animals were kept into polypropylene cages during the study protocol. The mice have proper access to dry feed and water *ad libitum* along with 12-h light/dark cycle at the temperature 25 ± 2°C, and the standard house conditions were maintained according to the CPCSEA guidelines.

#### Experimental Protocol

The animals were divided in seven groups (*n* = 5) and were given free access to feed and water for 1 week prior to dosing. In control group (1), the normal saline was administered, and scopolamine (0.4 mg/kg, i.p.) was injected to group 2 mice, Donepezil (3 mg/kg, i.p.), positive control, was given to the animals of group 3. The various extracts (150 mg/kg) were orally administered to animals of group 4–7 for 14 days. On 15th day, the scopolamine (0.4 mg/kg, i.p.) was given to the animals of group 3–7, and the protective effect against scopolamine induced amnesia was evaluated by following parameters.

##### Evaluation of Behavioral Parameters

The learning and memory potential of mice in different groups was evaluated by behavioral studies using elevated plus maze and passive avoidance apparatus. In elevated plus maze model, a wooden plus shape block comprised of open and closed arm was kept on an elevated place (50 cm from floor). The mice were placed toward the open arm of the elevated plus maze and transfer latency (TL) [time taken by mice to move inside the closed arm was recorded for 5 min (19)]. Passive avoidance is generally utilized to explain an experimental procedure in particular behavior of suppression to avoid noxious events learned by animals. Step down latency (SDL) is the time taken by the animal from steel grid floor to the wooden grid floor. In passive avoidance apparatus, electric shocks at 60 V at a frequency of 1 Hz for 0.5 s were given at steel grid floor. Each animal was kept at wooden floor in center during training periods. Shock was applied to steel grid floor. The time of animals step down from steel grid floor was recorded and kept its all paws on the wooden grid floor located in center. After 24 h of training periods for each animal, every animal was placed on the wooden grid floor and recorded the SDL of passive avoidance behavior for 5 min (9).

##### Estimation of Biochemical Parameters

On 17th day of experimental protocol, the whole brain of mice of each group was isolated and cleaned with ice cold normal saline after the 17th day of experimental protocol. The brain samples of mice were homogenized in the phosphate buffer (pH 7.4) and instantaneously centrifuged at 10,000 rpm for 15 min. The brain homogenate of animal was used to examine the various biochemical parameters. The Aebi method was employed to carryout catalase activity (20). For this, the reaction mixture was prepared using 100 μl of brain homogenate, 0.1 mM phosphate buffer (pH 7.4), and 30 mM hydrogen peroxide (1 ml). The change in absorbance was noted by UV-visible spectrophotometer at 240 nm, and the activity was expressed in nmol of H_2_O_2_ consumed/min/mg/protein. According to Ellman method, the glutathione activity was analyzed (21). Briefly, 10% of trichloroacetic acid was mixed into equal quantity of brain homogenate. The mixture was centrifuged for 15 min to separate out the proteins present in it. Later, the supernatant of the above mixture (0.01 ml) was mixed with 2 ml phosphate buffer (pH 8.4) and required amount of 0.5 ml 5'5-dithiobis (2-nitrobenzoic acid) along with double distilled water (0.4 ml). The mixture was stirred vigorously and its absorbance was recorded within 15 min at 412 nm. The reduced glutathione concentration was indicated by unit μmol/mg tissue. Griess reagent was utilized to evaluate nitric oxide content in brain homogenate by calculating total nitrate/nitrite (NOx) (22). Vanadium trichloride is responsible for chemical conversion of nitrate to nitrite. In this reaction, chromophores are formed by diazotization of sulfanilamide due to acidified nitrite binding along with N-(1-naphthyl) ethylenediamine to obtain colored azo derivative which was measured at 540 nm using spectrophotometer and the unit was expressed as μmol/mg tissue. The superoxide dismutase (SOD) concentration was also evaluated in brain homogenate of animals of different groups. For this, the mixture of n-butanol (4 ml) and brain homogenate was stirred vigorously. The reaction mixture was kept aside for few minutes and later on, n-butanol layer was separated by centrifugation for 15 min. The chromogen's color intensity was recorded against n-butanol using spectrophotometer at 560 nm (23).

##### Acetylcholinesterase Inhibitory Potential

The acetylcholinesterase (AChE) inhibitory potential of different extracts was also evaluated in brain homogenates by Ellman method (24). Briefly, the reaction mixture was prepared using 0.4 ml brain homogenate, 0.1 M phosphate buffer (pH 8), and 0.01 M dithio-bis-nitro-benzoic acid (0.1 ml). The mixture was incubated for 5 min at room temperature, acetylthiocholine iodide as a substrate was added, and absorbance (at 412 nm) was recorded using spectrophotometer for 5 min.

#### Statistical Analysis

All results were observed in triplicate, and the significance of the data was evaluated by analysis of variance (ANOVA) using GraphPad Prism 9.0 software.

## Results and Discussion

*Celastrus paniculatus* Willd commonly known as the “*Jyotishmati*” and is considered to be as “elixir of life” in the treatment and prevention of various diseases. Seed powder and oil are tremendously utilized in commercial ayurvedic formulations for the improvement of learning and memory. The CEMs such as maceration and percolation was mostly used in industries to prepare the extracts of the seeds of the selected herb. Looking at the potential medicinal and commercial significance of the selected plant, and the limitations of the traditional methods, it is decided to prepare the extract by green methods such as MAE and UAE. Also, the various extraction parameters in these methods were optimized using BBD coupled with RSM. Our study also aimed to comparatively evaluate the different extracts for possible improvement in EY, recovery of TPC, TFC, antioxidant, AChE inhibitory, and memory enhancing potential. The coarsely powdered seeds of selected plant were extracted by conventional methods, and the EY (%) was found to be 12.53 and 15.93%w/w in maceration and percolation techniques, respectively.

### Microwave-Assisted Extraction of Herb

Microwave-assisted extraction is one of the effective and promising green extraction technique based on electromagnetic radiations. In this, the high-speed energy radiations quickly heat up the plant material and solvent leading to efficient extraction process (25). In MAE, improved ionic conduction and dipole rotation in the solvent molecules facilitate the effective recovery of desired molecules in plant material (25–27). Various parameters such as microwave power, solvent concentration, irradiation time, solvent nature, solvent/solid ratio, extraction temperature, and size of particle significantly alter the extraction efficiency and yield of phytoconstituents and thus need to be optimized (25).

For the MAE of selected herb, the temperature was selected based on some preliminary experiments. It was reported that higher temperature had positive effect on EYs, but it cannot be increased indefinitely. Also, several studies in past for MAE used the temperature in the range of 60–80°C (28, 29). Ahmad et al. (30) reported that increased temperature that is too high can cause damage to sample matrix and can also lead to change in structure of targeted compounds (31). Keeping in view the above facts and results of some preliminary experiments, we selected the temperature of 80°C for MBE of CP. Further, the literature study about the MAE of herbs indicated that absorption of microwave energy by the extractant is one of the main factors responsible for the possible intensification of extraction process (32, 33). Green solvents with high dielectric constant (ε') such as ethanol (ε'-25) helped to absorb the microwave energy efficiently and dissipate it as heat to the surrounding solvent (34). Moreover, the water (ε'-80) is added in the ethanol to some extent to improve the dielectric constant of the solvent for MAE (35). The addition of water increases polarity indices of organic solvent which reflect as an easy absorption of microwave energy and thus increasing the temperature inside the sample leading to rupture of cell walls and causes the faster release of phytoconstituents (29, 36–39). Hence, in this research, the ethanol was selected for MAE of selected herb, and the concentration of water in the ethanol was optimized using the design protocol.

### Model Fitting for MAE

**Figure 1:**
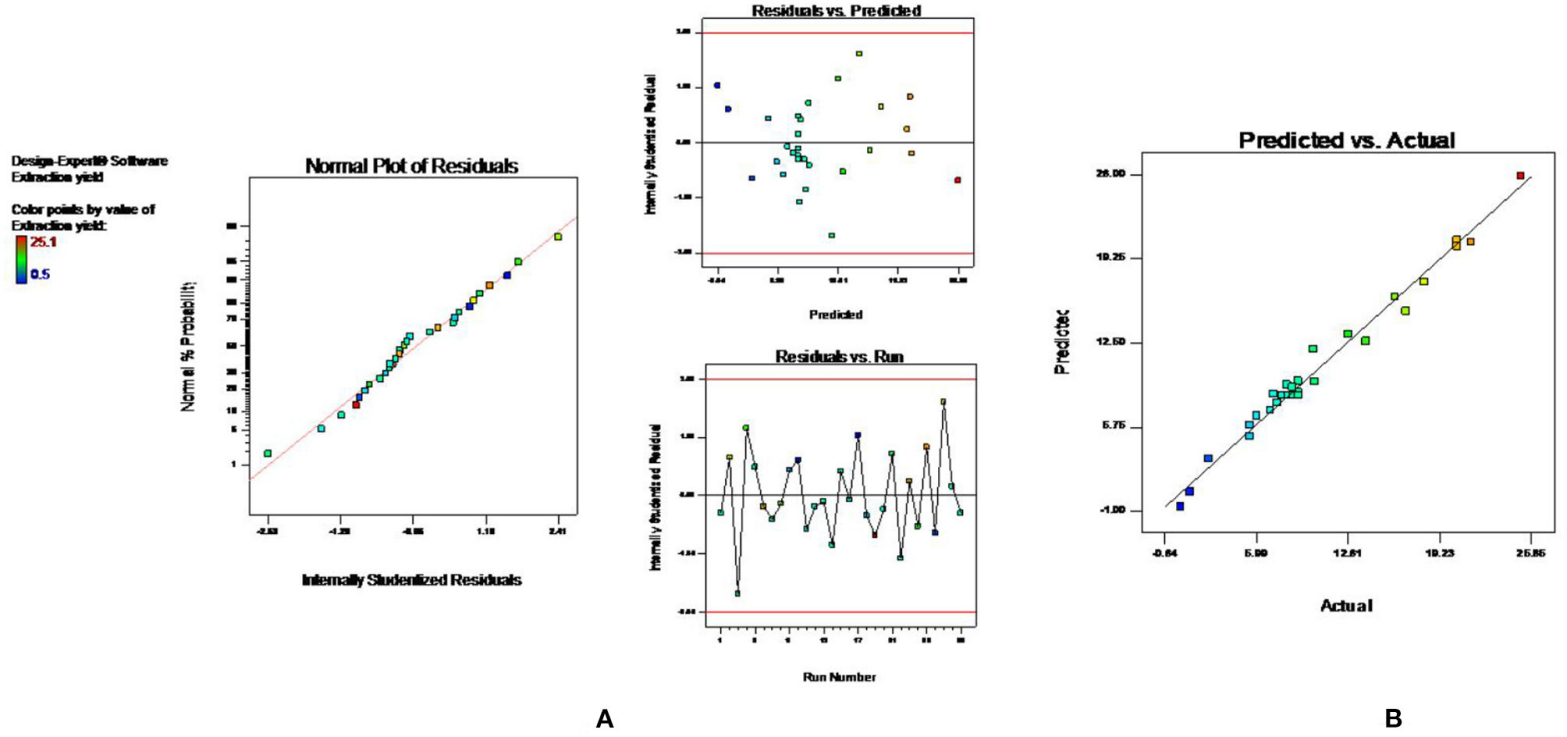
Diagnostic plots to confirm the fitness of developed model in MAE **(A)** normal plot of residual **(B)** predicted vs. actual value.

The effect of other variables such as microwave power (X_1_ 100–300 W), time (X_2_ 1–10 min), solvent concentration (X_3_ 20–80%), and solvent to drug ratio (X_4_ 5–50 ml/g) on percentage yield of extract (Y_1_) was studied using BBD coupled with RSM. A total of 29 experimental runs, as suggested by the design, were carried out and the results are depicted in Table 1. The experimental data were evaluated by analysis of variance to know whether the model is statically significant or not. The F value (58.18) exhibited by model indicated that model was significant. Also, the lower probability value (*p* < 0.0001) also signifies that the model is extremely significant, and the difference in response can be explained by the regression equation. The R^2^ (0.96) value estimates the degree of fitness and basically is the ratio of explained to total variance. The vicinity of R^2^ to unity indicated the better fitness of developed model along with actual data. Also, the high value of predicted R^2^ (0.90) confirmed the better correlation of independent variables with the selected response. In addition, the low value of coefficient of variance (10.83) and high adequate precision (32.408) confirms the reliability of data and precision to noise ratio of justified the fitness of developed model to navigate the design space. The fitness of the developed model was also justified by the diagnostic plots (Figure 1). The normal plot of residual (Figure 1A) indicated that no significant variations are there and it is normally distributed. Also, the predicted values present near the actual values as indicated in Figure 1B (13). Further, a second-order polynomial mathematical equation (1) was also derived using multiple regression analysis of the obtained data.


$$\begin{array}{l} \left(Y\right)=1052.33+5.60X_{1}+12.61X_{2}+267.91X_{3}+286.16X_{4}\\ +4.84X_{1}X_{2}+3.42X_{1}X_{3}+34.22X_{1}X_{4}+27.56X_{2}X_{3}\\ +34.81X_{2}X_{4}+\;48.30X_{3}X_{4}+9.13X_{1}^{2}+0.59X_{2}^{2}+257.38X_{3}^{2}\\ +20.74X_{4}^{2} \end{array}$$

**Table 1:** Actual and predicted EY at different extraction conditions in MAE.

**Run**	**Microwave power (Watt) (X_1_)**	**Time min (X_2_)**	**Solvent conc. (%) (X_3_)**	**S/S Ratio ml/gm (X_4_)**	**% Extraction yield (Y)**	
					**Actual yield (w/w)**	**Predicted yield (w/w)**
1	200.00	5.50	50.00	27.50	7.8	8.26
2	200.00	1.00	80.00	27.50	18.1	17.38
3	300.00	1.00	50.00	27.50	10.1	11.95
4	300.00	5.50	20.00	27.50	13.9	12.63
5	200.00	5.50	50.00	27.50	9	8.26
6	100.00	5.50	80.00	27.50	20.5	20.71
7	200.00	5.50	20.00	50.00	9	9.45
9	100.00	5.50	50.00	5.00	5.5	5.02
10	300.00	5.50	50.00	5.00	1.2	0.53
11	200.00	5.50	20.00	5.00	6	6.64
12	300.00	10.00	50.00	27.50	7.5	7.70
13	200.00	5.50	50.00	27.50	8.1	8.26
14	200.00	5.50	80.00	5.00	8.2	9.14
15	100.00	10.00	50.00	27.50	9	8.54
16	200.00	10.00	50.00	50.00	7	7.08
17	200.00	1.00	50.00	5.00	0.5	0.4
18	200.00	10.00	20.00	27.50	5.5	5.88
19	200.00	5.50	80.00	50.00	25.1	25.85
20	200.00	5.50	50.00	27.50	7.9	8.26
21	100.00	5.50	20.00	27.50	10.2	9.41
22	100.00	1.00	50.00	27.50	7.2	8.39
23	300.00	5.50	80.00	27.50	20.5	20.23
24	200.00	1.00	20.00	27.50	12.6	13.18
25	200.00	10.00	80.00	27.50	21.5	20.58
26	200.00	10.00	50.00	5.00	2.5	3.21
27	200.00	1.00	50.00	50.00	16.8	15.03
28	200.00	5.50	50.00	27.50	8.5	8.26
29	100.00	5.50	50.00	50.00	8.6	8.93

**Figure 2:**
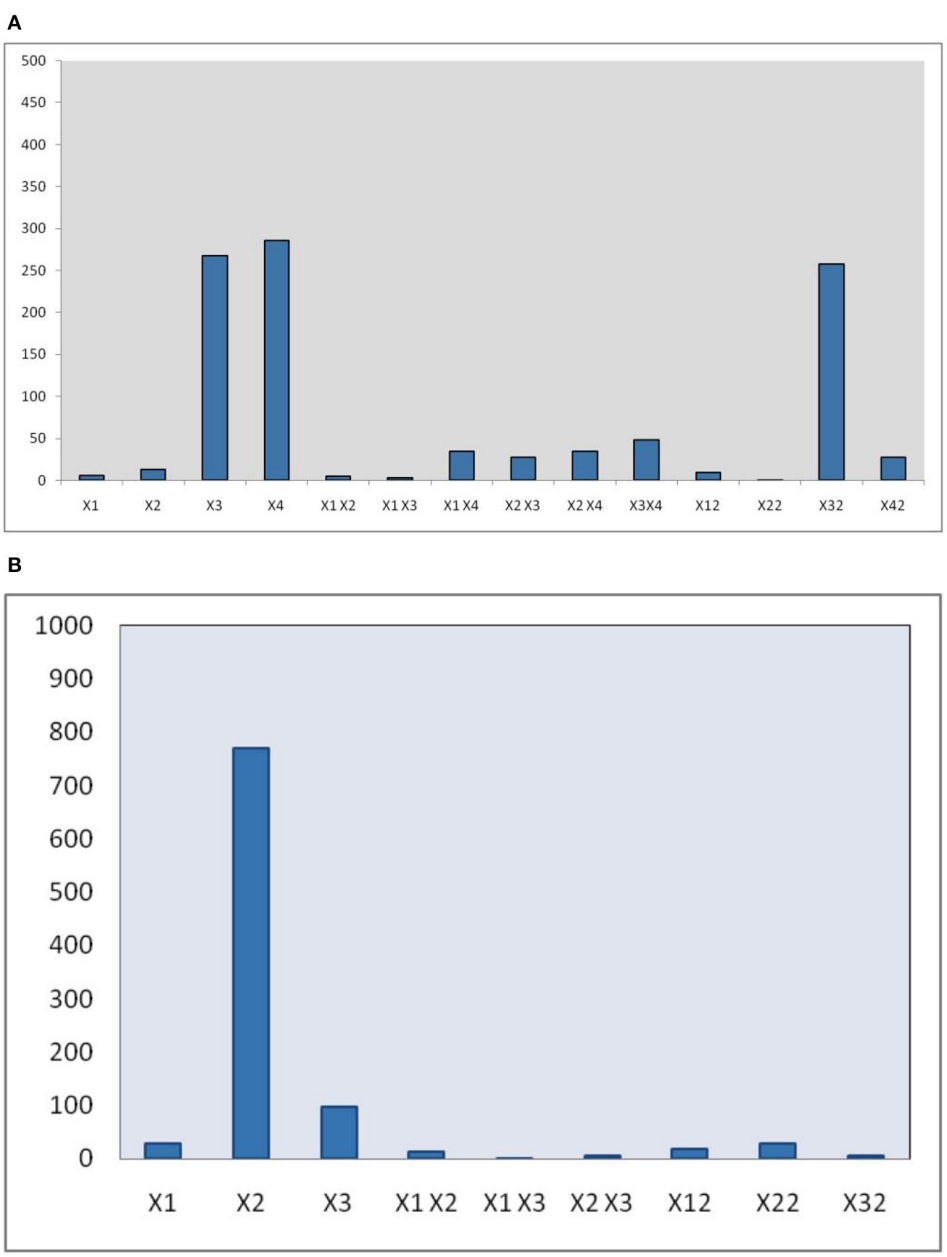
Pareto chart indicating magnitude of different variables/terms on EY **(A)** MAE **(B)** UAE.

The developed polynomial equation suggested that the linear (X_2_, X_3_, and X_4_), interactive (X_1_X_4_, X_2_X_3_, X_2_X_4_, and X_3_X_4_), and quadric terms (${\text{X}}_{1}^{2}$, ${\text{X}}_{3}^{2}$, and ${\text{X}}_{4}^{2}$) are highly significant (*p* < 0.01). Also, the pareto chart (Figure 2A) represnted the magnitude of various terms on the EY.

### Response Surface Analysis

**Figure 3:**
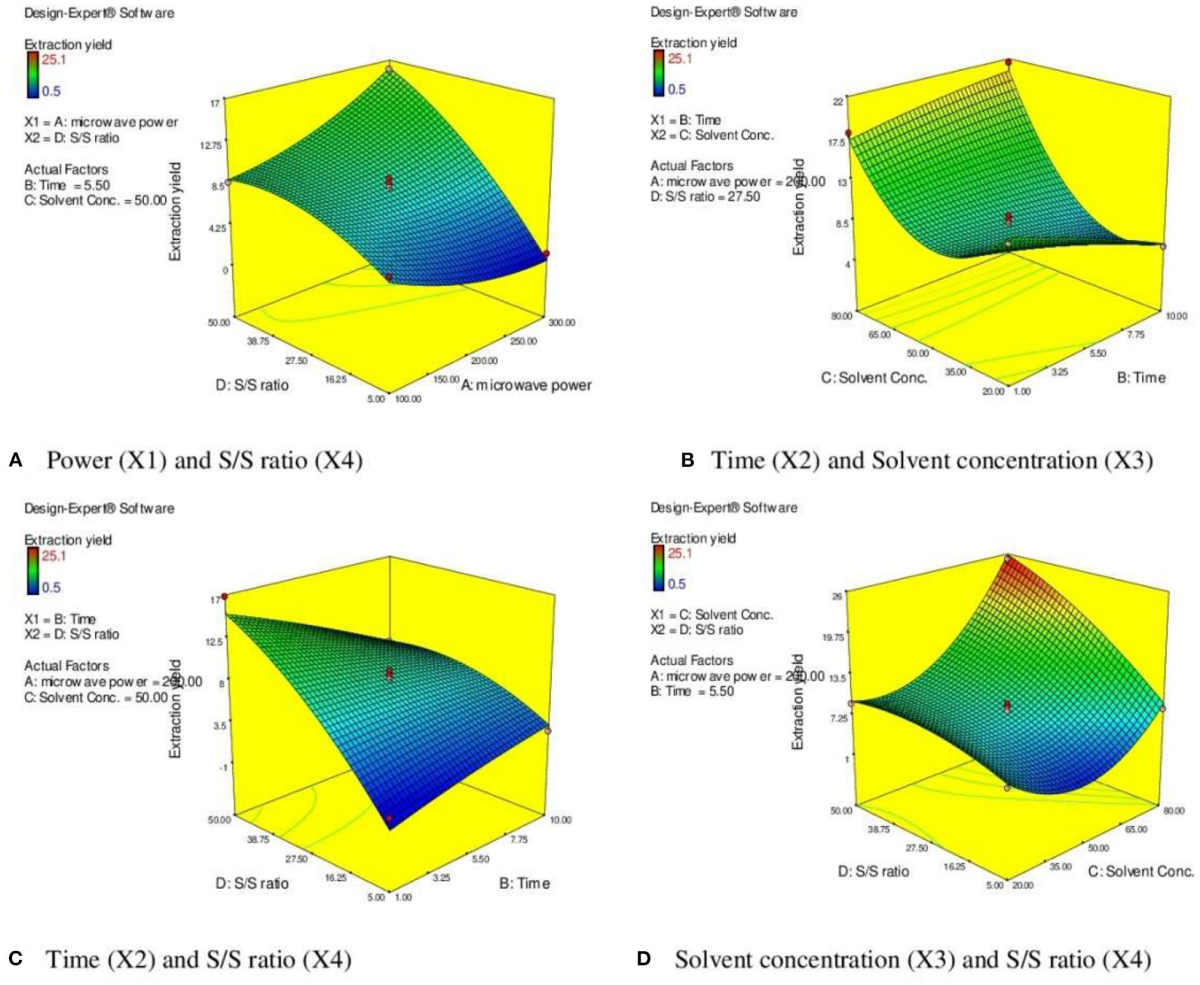
3 D diagram indicating the effect of different variables (X_1_, X_2_, X_3_, X_4_) on the response, % EY (Y) in MAE. **(A)** Power (X1) and S/S ratio (X4). **(B)** Time (X2) and Solvent concentration (X3). **(C)** Time (X2) and S/S ratio (X4). **(D)** Solvent concentration (X3) and S/S ratio (X4).

From response surface analysis Figure 3A, it was evident that microwave power (X_1_) and solvent to drug ratio (X_4_) for particular time (5.50 min) and solvent concentration of 50% produced the positive significant effect on EY (p <0.01). Microwave power has a significant effect on EYs. Higher EY recovered at the 200 watts, and after that, there was a decrease in the EYs up to 300 watts. When irradiation extends to more than 5 min, the EY starts to decrease. Moreover, higher power and longer irradiation time can also damage the structure of desired compounds (40). Degradation of plant active can also be observed at higher microwave power and increased irradiation time in MAE (30, 31, 41, 42).

Response surface analysis (Figures 3B,C) indicated the rise in irradiation time significantly enhanced the EY (p <0.001) to certain limit (5.5 min). The resistance of solution to electrophoretic migration of ions would eventually cause quick heating of plant material within a solvent mixture. The cell walls of plant matrix were ruptured due to the sudden increases in the temperature of a solvent mixture, reinforcing cavitations, and turbulence effects that occurred through microwave power, and further mass transfer improved (43–46). After this, there is no significant alteration in the response parameter (EY). Moreover, the prolong irradiation of plant material in MAE could decrease the concentration of target compounds due to heating the plant materials at high temperature. It is evident from the literature that when irradiation continued to increase, then after certain time, the EYs began to fall. Higher extraction time under microwave radiation could induce degradation of active constituents resulting in reduction of EYs (47, 48).

Figure 3D represents the effect of solvent concentration (X_3_) and solvent to solid ratio (X_4_) on the EY. When ethanol concentration increased from 20 to 80%, the EY enhanced significantly (p <0.001). The efficiency and selectivity of MAE is based on the dielectric constant of the solvent mixture. Optimizing the amount of water (20%) in the solvent significantly improves the dielectric constant of menstruum and reflects in easy absorption of microwave energy leading to rupture of cell walls of plant matrix and results in improved EY (29, 36, 37, 49). The solvent/solid ratio is other important parameter that influences the extraction process. The large solvent/solid ratio is beneficial for better mass transfer and dissolution of solutes (50, 51). This study also supports the positive effect of higher S/S ratio (41 ml/g) but further increase in the ratio did not significantly improve the EY.

### Ultrasound-Assisted Extraction

These days, UAE is effectively employed for the extraction of herbs owing to its improved and better results with a green approach. The acoustic cavitation effect produced by the ultrasound waves in the drug particles is mainly responsible for the upgraded diffusion of menstruum and results in improved EY (3). UAE is considered to be the green technique that can provide high reproducibility in small time and low energy inputs (52–56). The various parameters such as solvent/solid ratio, sonication time, ultrasonic power, amplitude, and temperature affect the extraction efficiency in UAE and need to be optimized for improved yield (57, 58). Based on the literature study and results of our previously published paper, we can say that the UAE is successful at lower temperature (40–70°C) and low sonication frequency (20–30 KHz) (3, 59). Therefore, for this study, the experiments were carried out at 24 KHz and at 70°C. Also, the interval pulse modulation can prevent maximum exposure to heating in the sample mixture and can offer improved results. Hence, the pulse of 0.5 s at maximum intensity was selected based on the results from preliminary experiments. Moreover, the sample is sieved through the 60 mesh size for the optimum and efficient mass transfer process necessary for the improved yield in UAE (60). For this research, the other parameters for UAE of selected herb such as sonication time, solvent/solid ratio and solvent concentration (30–90%) are optimized using BBD coupled with RSM. A total of 17 experiment runs on different extraction conditions, as suggested by the design, were conducted and actual or predicted EY (Y) was calculated (Table 2).

**Table 2:** Actual and predicted EY at different extraction conditions in UAE.

**Run**	**(X_1_) Sonication time min**	**(X_2_) Solvent conc. %**	**(X_3_) Volume of solvent ml**	**% Extraction Yield (Y)**	
				**Actual (w/w)**	**Predicted (w/w)**
1	5.00	50.00	20.00	8.41	8.66
2	12.50	90.00	30.00	37.88	37.53
3	12.50	70.00	20.00	26.90	26.94
4	5.00	70.00	30.00	24.9	24.81
5	12.50	70.00	20.00	26.39	26.94
6	12.50	70.00	20.00	26.81	26.94
7	20.00	70.00	10.00	21.55	21.64
8	12.50	50.00	10.00	10.61	10.97
9	20.00	50.00	20.00	16.65	16.21
10	20.00	70.00	30.00	28.72	29.32
11	5.00	90.00	20.00	31.61	32.05
12	5.00	70.00	10.00	19.18	18.59
13	12.50	70.00	20.00	26.94	24.94
14	12.50	50.00	30.00	15.74	15.59
15	20.00	90.00	20.00	32.29	32.05
16	12.50	70.00	20.00	27.66	26.94
17	12.50	90.00	10.00	28.09	28.25

### Model Fitting for UAE

**Figure 4:**
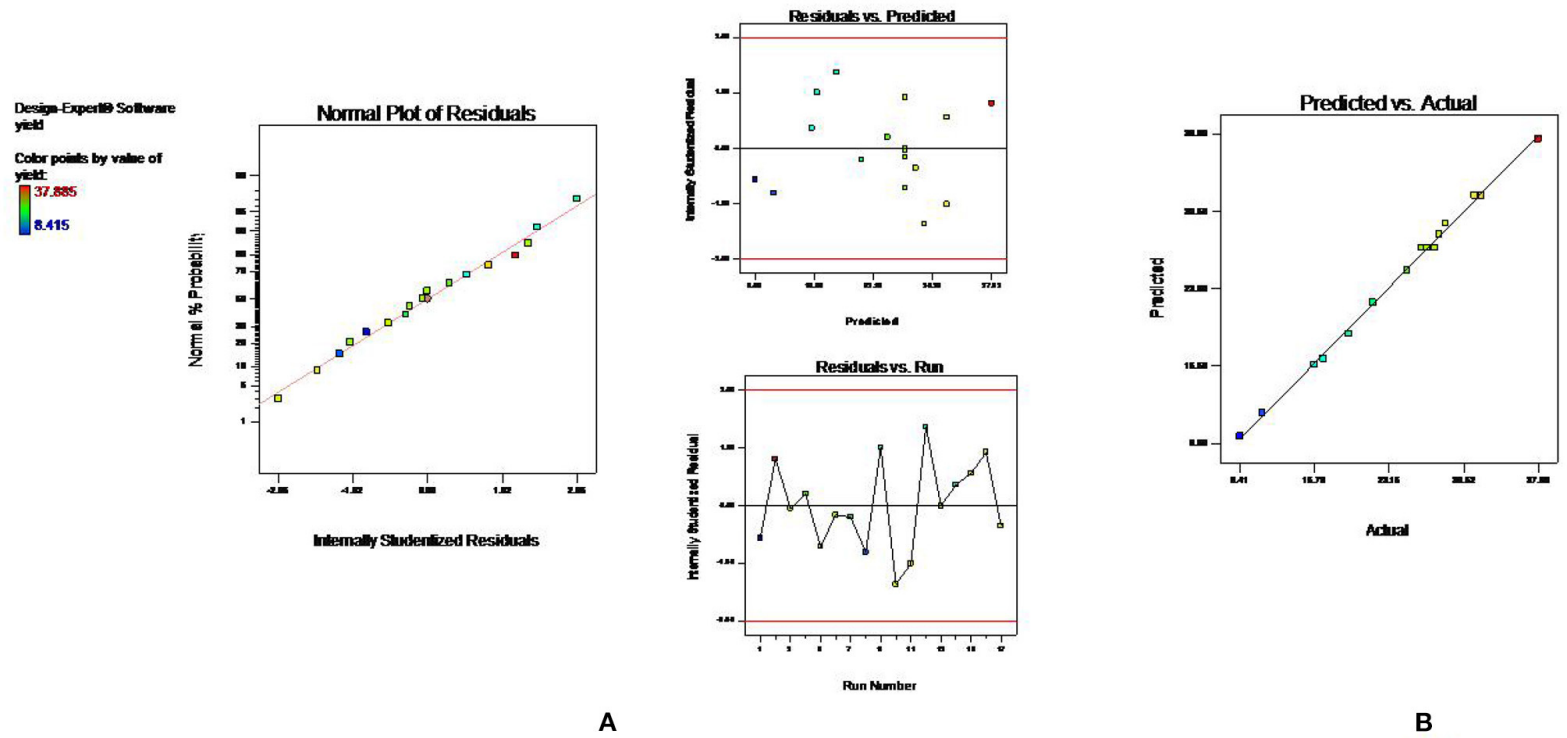
Diagnostic plots to confirm the fitness of developed model in UAE **(A)** normal plot of residual **(B)** predicted vs. actual value.

The statistical significance of the experimental data was analyzed, and it was found that developed model is highly significant (*p* < 0.001) with the high F value. The low *p*-value also indicated that the variation in response can be efficiently explained by the developed regression equation. Also, the value of coefficient of determination, R^2^, and adjusted R^2^ near to 1 denoted high degree of the correlation and better fitness of model along with actual data. Moreover, the adequacy in precision (64.83) and coefficient of variance (2.4) further confirms the fitness of developed model to analyze the results. Model fitness was further approved by normal plot of residual (Figure 4A), which confirmed that no significant deviation of variance is present. Moreover, the vicinity of predicted value to the straight line in Figure 4B confirmed its fitment with actual data (13). Multiple regression analysis of data is used to derive the second-order polynomial equation (Equation 2) which significantly determines the relationship between independent parameters and response (Y).


$$\begin{array}{l} \left(Y\right)=974.24+28.54X_{1}+769.50X_{2}+96.67X_{3}+14.25X_{1}X_{2}\\ \;+0.53X_{1}X_{3}+5.43X_{2}X_{3}+18.52X_{1}^{2}+28.51X_{2}^{2}+6.63X_{3}^{2} \end{array}$$

The statistical analysis of the data further confirms that the all linear terms (X_1_, X_2_, and X_3_), interactive terms (X_1_X_2_, X_2_X_3_), and quadric terms (${\text{X}}_{1}^{2}$, ${\text{X}}_{3}^{2}$, and ${\text{X}}_{3}^{2}$) are highly significant (*p* < 0.01), whereas the coefficient of interaction term (X_1_X_3_) is not significant (*p* > 0.05). Moreover, the magnitude of these different terms on EY also represented as a pareto chart in Figure 2B.

### Response Surface Analysis in UAE

**Figure 5:**
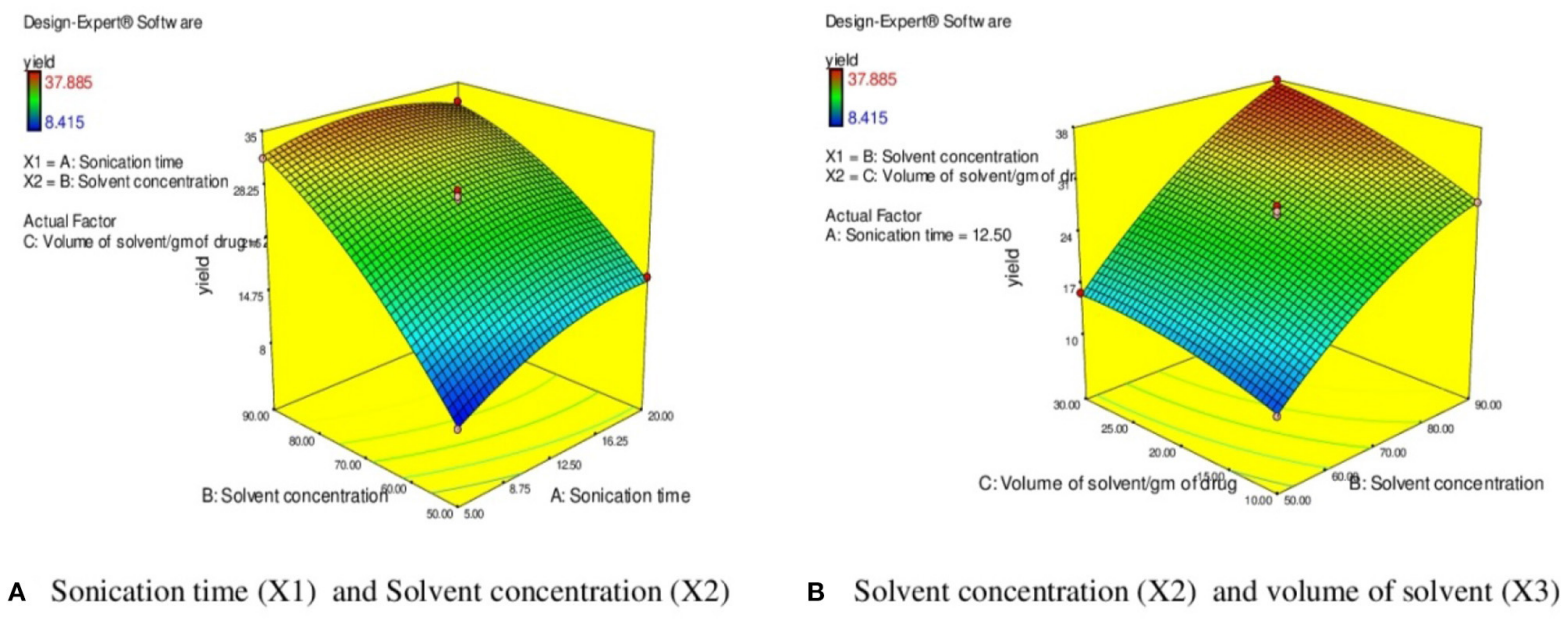
3 D diagram indicating the effect of different variables (X_1_, X_2_, X_3_) on the response, % EY (Y) in UAE. **(A)** Sonication time (X l) and Solvent concentration (X2). **(B)** Solvent concentration (X2) and volume of solvent (X3).

The effect of sonication time (X_1_) at a fixed volume of solvent (20 ml) on EY can be evident from Figure 5A. It was observed that the EY enhanced till 12.5 min and followed by a decline in yield of extract with further increase in the extraction time. It was observed that EYs increase with rising in the extraction time which followed by a decline in yield with further increase in the extraction time. On extraction with sound waves, the plant matrix is completely cracked and due to rupture of cells, the insoluble substances and cytosol within vessels get suspended in extraction liquid and resulting in enhanced yield (60). However, extended exposure to sonication resulted in decomposition and degradation of active constituents and causes the low yields (57, 58). Similarly, the high solvent/solid ratio (30 ml/g) increased the EYs due to higher interaction between sample and solvent which improved the solubility of active constituents and the better penetration of the plant matrix further results in maximum EYs (Figure 5B) (57). Further increase in the liquid/solid ratio does not influence the extraction efficiency but diminished ultrasonic energy and could result in reduced EYs (61). Moreover, Figure 5B depicts the response surface analysis, and it was found that interactive effects of solvent concentration (X_2_) and volume of solvent (X_3_) at fixed sonication time (12.50 min) had significant effects on EY (*p* < 0.05).

In this research, different extraction factors in MAE and UAE were optimized using the BBD coupled with RSM to obtain maximum EY (%). The numerical optimization approach was applied, and the microwave power (268.91 W), time (6.83 min), solvent concentration (80%), and solvent to solid ratio of (41.43 ml) were predicted as optimized extraction conditions in MAE to achieve the maximum EY (25.1 ± 1.5% w/w). Similarly, optimized conditions for UAE was predicted to be as sonication time (11.81 min), solvent concentration (90%), and volume of solvent (27.36 ml) to achieve maximum EY of 36.5 ± 2.3% w/w. The plant material was also extracted at the optimized conditions, and the EY was found to be in 95% confidence limit which validates the developed model.

### Estimation of TPC and TFC

The various extracts obtained by CEM are termed as MCP (maceration), SXCP (Soxhlet), OMCP (optimized extract of MAE), and OUCP (optimized extract of UAE) are also evaluated for the total phenolic and flavonoid content (Table 3). The TPC was significantly enhanced, *p* < 0.01, in green methods to 56.5 ± 3.1 in OUCP and 57.5 ± 2.8 mg GAE/g in OMCP from 13.33 ± 0.76 in MCP to 21.16 ± 1.52 mg GAE/g in SXCP. Also, the content of total flavonoid was intensified significantly (*p* < 0.01) from 8.16 ± 0.57 in MCP and 11.66 ± 0.76 mg RUE/g in SXCP to 26 ± 1.8 in OUCP and 28.5 ± 1.1 mg RUE/g in OMCP. Observations indicated that the unconventional methods not only improved the concentration of TPC and TFC but also drastically reduced the extraction time from 8 days (MCP) and 9 h (SXCP) to few minutes in MAE and UAE of selected herb at optimized conditions. These results are in concomitant to previous studies in which MAE and UAE significantly improved the yield of extract and plant actives in lesser time as compared to conventional methods (62, 63). Moreover, it has been reported that the extraction of phenolic compounds is a mass transfer process, and in past, MAE and UAE have also been proved to increase the mass transfer rate during the extraction process (25). Hence, the intensification of TPC and TFC could be attributed to enhanced positive effect on the mass transfer rate during the MAE and UAE of *CP*.

**Table 3:** Comparative evaluation of different extracts of *CP* Willd for EY, TPC, and TFC.

**Extract**	**Power (in Watt)**	**Time**	**Solvent to solid ratio (mL/g of drug)**	**Solvent concentration (%)**	**EY (%, w/w)**	**TPC (mg of GAE/g of extract)**	**TFC (mg of RUE/g of extract)**
OMCP	268.9	6.83 (min)	41.43	80	25.1 ± 1.5	57.5 ± 2.8	28.5 ± 1.1
OUCP	—–	11.81 (min)	27.31	90	36.5 ± 2.3	56.5 ± 3.1	26 ± 1.8
SXCP	300	9 (Hr)	50	100	15.93 ± 0.9	21.16 ± 1.52	11.66 ± 0.76
MCP	……	8 days	50	100	12.53 ± 0.8	13.33 ± 0.76	8.16 ± 0.57

### Scanning Electron Microscopy

**Figure 6:**
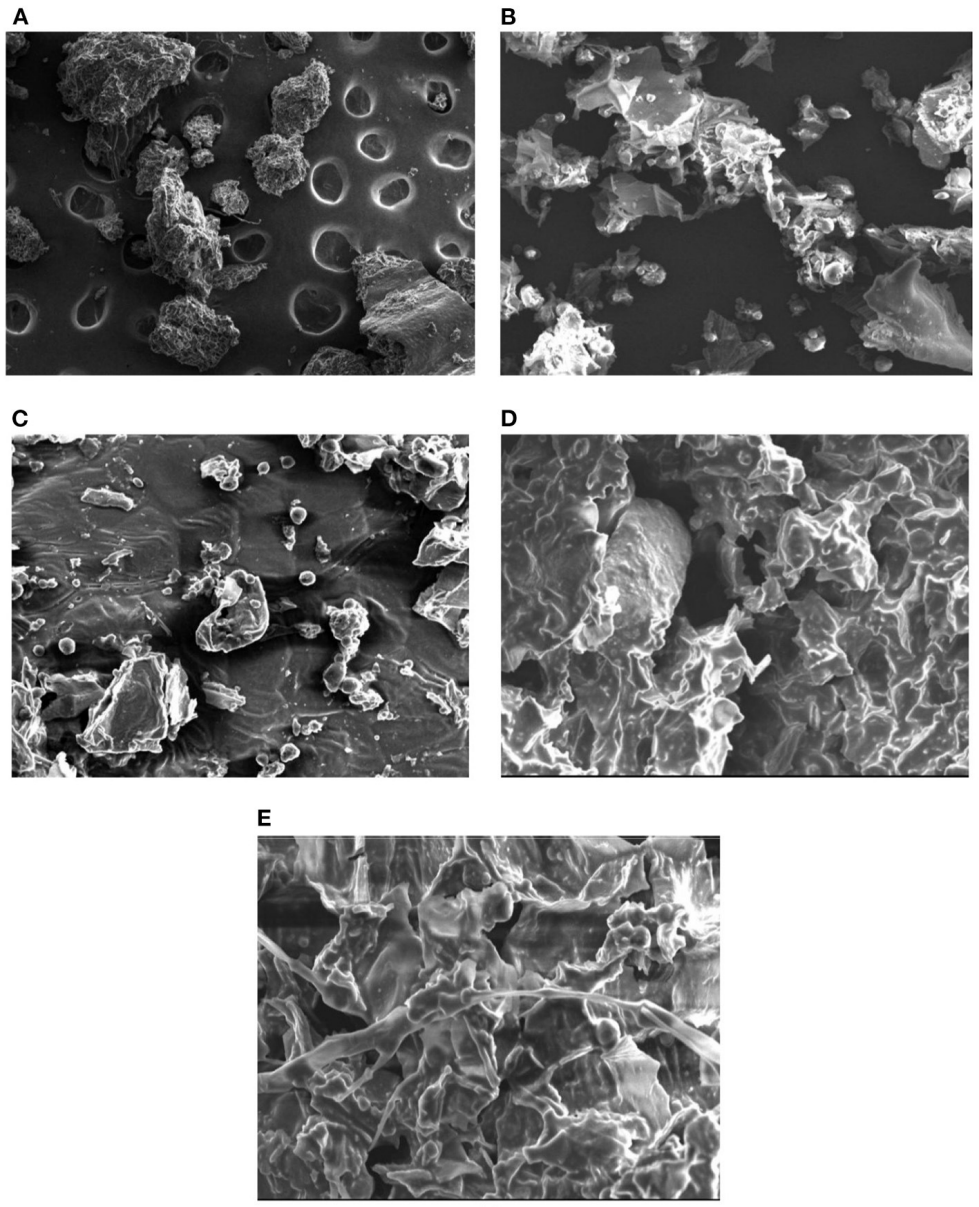
Scanning electron micrographs of different samples **(A)** untreated **(B)** after maceration **(C)** after percolation **(D)** after UAE **(E)** after MAE.

Generally, extraction process involves the different steps such as softening of herb by the solvent, permeation of menstruum, and solubilization of plant actives. The microstructure of herbal raw material is affected in different ways during the various extraction processes. The SEM of plant material after maceration and percolation (Figures 6B,C) indicated the herb particles gets flattened and become disorganized as compared to untreated samples (Figure 6A). The microstructures of raw material proved that in CEM, the extraction simply takes place through diffusion and solubilization process (64). The SEM analysis of marc left after UAE indicated the presence of several corrugations and lesions on the flat particles surface (Figure 6D). The shear force of sound waves could be responsible for such destruction in the morphology of herb particles. Moreover, the cavitation effect produced by the high-speed jet of the solvent can also cause the fragmentation of plant sample (3). Also, in past, researchers had also proved the erosive effect of sound waves (above 20 KHz) on the morphology of basil leaves (65). In Figure 6E, shrunk and crumbled surface of plant material was observed after MAE. This destruction could be attributed to extensive caused by microwave radiations. Also, the water present inside cell absorbed the microwave energy and creates high pressure which in turn resulted in crumbled surface of herb particles (63, 66).

### GC-MS Analysis

**Figure 7:**
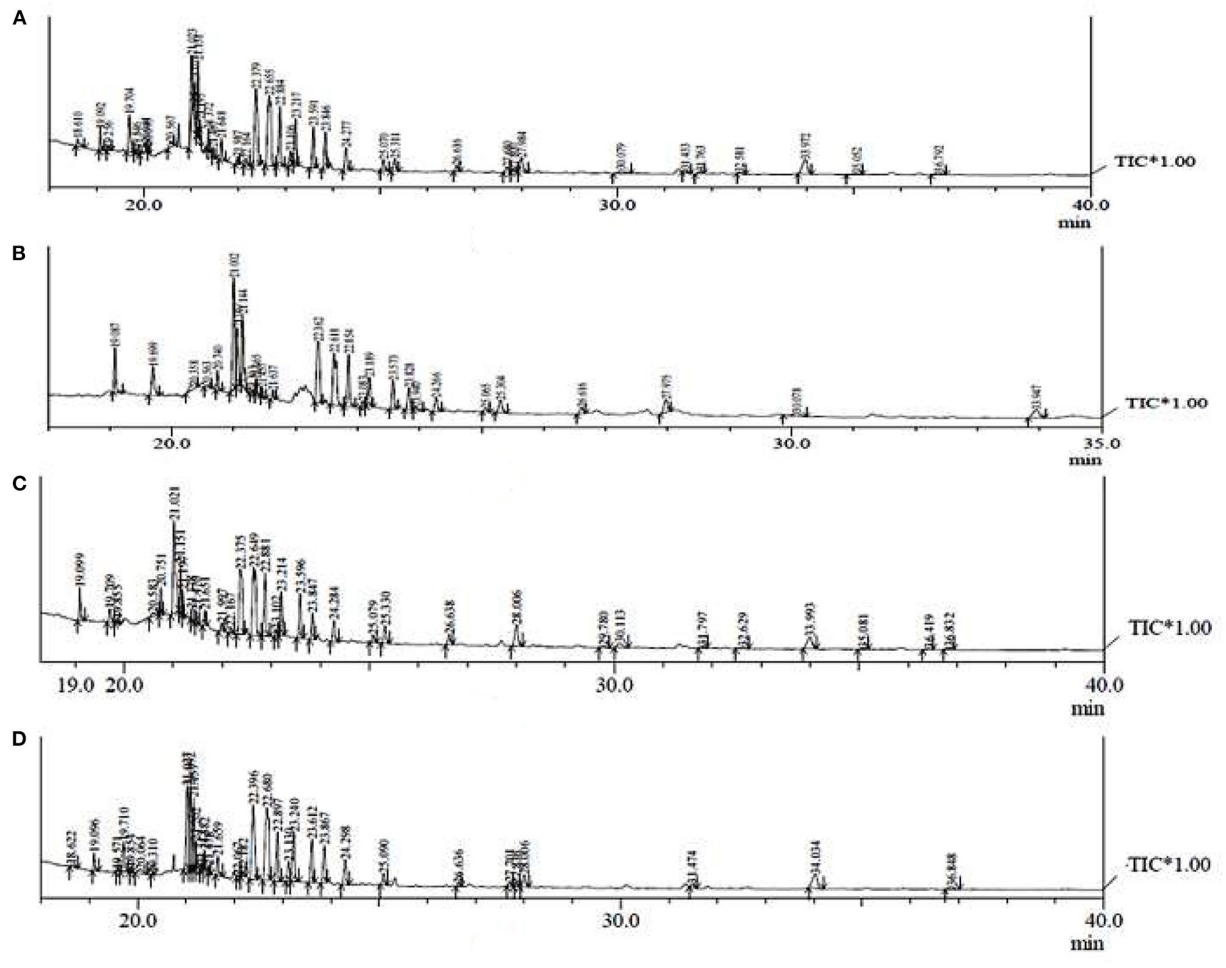
GC-MS chromatogram of different extracts **(A)** MCP **(B)** SXCP **(C)** OMCP **(D)** OUCP.

The GC-MS analysis of different extracts (MCP, SXCP, OMCP, and OUCP) was carried out, and the chromatogram of different samples is indicated in Figure 7. The retention time and % area of different components are given in Table 4. The analysis led to identification of different category compounds such as palmitic acid, hexanoic acid (esters); cholan-24-oic acid (steroidal esters); sitosterol, 3,9-epoxypregn-16-en-14-ol-20-one, estradiol 17-benzoate-3-p-phenylazobenzoate (steroids); (1S,4S,5S,6R,7R,9S,10S)-4,9-dibenzoyloxy-1,6,15-triacetoxy-dihydrto.beta agarofuran, Celorvicol (sesquiterpenes); and pilocarpine (alkaloid). The results confirmed that the most of the constituents present in MCP and SXCP are also present in OMCP and OUCP, and the concentration of some compounds such as palmitic acid, hexanoic acid, sitosterol, and pilocarpine is significantly enhanced in extracts obtained in green methods. Moreover, esters such as hexadecanoic acid and stearic acid are only present in OUCP whereas the steroidal compounds such as cholestane and bisnorandrostane are reported in OMCP. The observed results are concomitant to the previous reports in which MAE and UAE significantly improved the yield of plant actives. For instance, yield of ginsenosides from *Panax ginseng*, solanesol, a non-cyclic terpene from the tobacco leaves, total phenolics from *Ipomoea batatas*, and *Phaseolus vulgaris* and Camptothecin from *Nothapodytes foetida* was reported to get increased in MAE (63). Also, the UAE of seeds of *Isatis indigotica* significantly enhanced (up to 83%) the concentration of fatty acid esters (67). Further, the past scientific evidences proved the presence of secondary metabolites such as sterol, sesquiterpenes, and so on in the cytoplasm of cell (63, 68). Hence, the observed results could be attributed to the rupturing of cell membrane by the microwave radiations and cavitation effect produced by the sonication of plant material, which in turn oozes out the plant actives from the cytoplasm into the surrounding fluid. Moreover, esters such as hexadecanoic acid and stearic acid are only present in OUCP whereas the steroidal compounds such as cholestane and bisnorandrostane are reported in OMCP.

**Table 4:** Possible constituents reported in different extracts of *CP* Willd by GC-MS analysis.

**Chemical constituents**	**MCP**		**SXCP**		**OMCP**		**OUCP**	
	**R_T_ (min)**	**Area (%)**	**R_T_ (min)**	**Area (%)**	**R_T_ (min)**	**Area (%)**	**R_T_ (min)**	**Area (%)**
Palmitic acid	14.834	0.98	14.838	1.75	14.844	2.36	14.853	6.15
Hexanoic acid, pentadecyl ester	20.041	1.36	19.699	1.52	19.709	2.95	19.710	3.06
9-Octadecenoic acid (Z)-, hexadecyl ester	21.023	5.14	21.002	9.38	21.021	12.16	21.027	10.23
Squalene	21.151	3.89	21.144	5.27	21.151	3.05	….	….
Cholan-24-oic acid, 3-(acetyloxy)-12-oxo-, methyl ester, (3 alpha.,5 beta.)	21.372	1.04	21.365	0.87	21.021	12.16	21.382	1.15
3,9-epoxypregn-16-en-14-ol-20-one, 11,18-diacetoxy-3-methoxy	21.648	1.07	21.637	0.64	21.651	1.56	21.659	1.77
Tribenzoyl-d-mannosan	22.655	11.16	22.618	7.54	22.649	12.16	22.680	13.61
1,5-Anhydro-D-glucitol	23.106	1.08	23.083	0.46	23.102	0.58	23.130	2.15
Sitosterol	23.217	4.13	23.189	2.67	23.214	4.52	23.240	5.90
Lupan-3-ol, benzoate	23.591	3.63	24.266	1.43	23.596	4.44	23.612	4.50
(1S,4S,5S,6R,7R,9S,10S)-4,9-dibenzoyloxy-1,6,15-triacetoxy-dihydrto.beta.-agarofuran	23.846	3.16	23.828	2.05	23.847	2.63	23.867	4.07
Oleyl oleate	24.277	2.28	23.573	2.58	24.284	2.39	24.298	2.94
Ursa-9(11),12-dien-28-oic acid, 3-(acetyloxy)-, methyl ester, (3.beta.)	25.070	1.12	25.065	0.44			25.090	1.30
Pilocarpine	26.616	0.73	26.616	0.84	26.638	1.35	26.636	0.42
11-(benzoyloxy)-3-isopropyl-1,2,3,3A,4,5,6,6A,7,12-decahydrocyclopenta[D]anthracen-8-yl benzoate	27.984	1.99	27.975	1.86	28.006	3.75	28.006	2.42
N,1-Dibenzoyl-5-Amino-6-(Benzyloxy)-2-Methylindole-3-Carboxylic Acid Methyl Ester	33.972	3.04	33.947	1.92	33.993	3.17	34.034	3.60
6-Ethyl-3-decanol, TMS derivative	19.256	0.22	20.358	1.13				
Estradiol 17-benzoate-3-p-phenylazobenzoate	30.079	0.99	30.078	0.83	30.113	1.14		
1,3-cyclohexadecanedione					25.330	2.30	20.064	1.04
Celorvicol	27.800	0.20	-	-	-	-	27.836	0.35
O-benzyl-n,n-dibenzoyl-2,5-diamino-4-(1-(methoxycarbonyl)-2-oxopropyl)phenol	32.581	0.22			31.797	0.39	31.474	0.52
Pseudotigogenin diacetate	22.884	4.76	22.854	4.41	22.881	6.59	22.897	5.24

### Evaluation for Memory Enhancing Potential

The various extracts of seed powder of *CP* Willd obtained by CEMs and green methods at optimized conditions are also evaluated for memory enhancing potential in experimental mice.

#### Estimation of Behavioral Parameters

**Figure 8:**
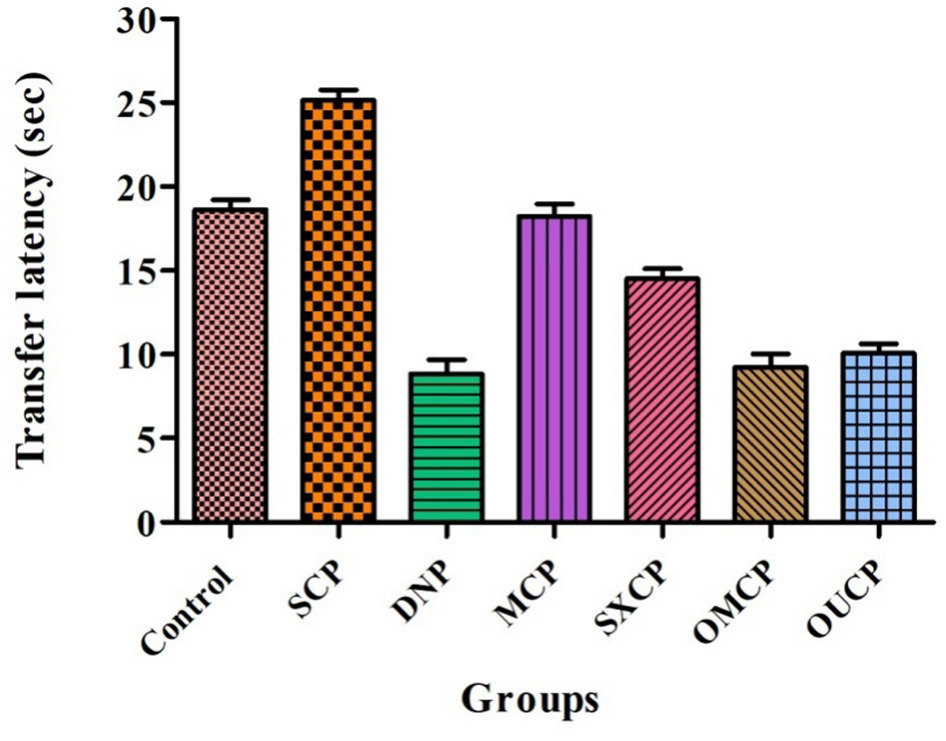
Effects of CP seed extracts on TL.

**Figure 9:**
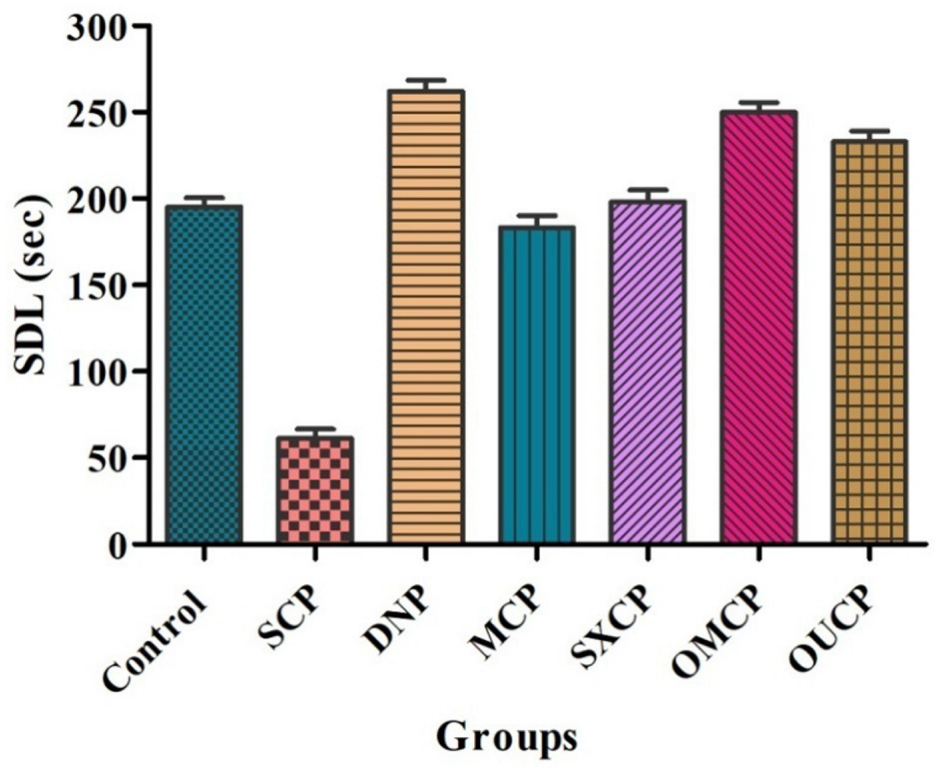
Effects of CP seed extracts on SDL.

Elevated plus maze was used to evaluate the retention of learning and memory and cognitive impairments in various optimized groups of different groups. Scopolamine caused short-term memory impairment in animals. TL (time taken by the animal from open arms to closed arms) was recorded at 15th day after the 24 h of drug administration for 5 min. The effects of different extracts of CP on TL reduced are represented in Figure 8. Scopolamine increased significantly TL as compared to control (*p* < 0.005). OMCP and OUCP alter the memory impairments significantly as compared to conventional extracts (MCP and SXCP, *p* < 0.01) and negative control group (SCP) (*p* < 0.05). Scopolamine also significantly decreases the SDL as compared to control group in different groups (*p* < 0.005). The SDL shown by the animals of different groups is represented in Figure 9. OMCP and OUCP reversed the effects of scopolamine in significant manner (*p* < 0.05). OMCP significantly increased SDL (*p* < 0.01) as compared to CEMs (MCP and SXCP). Also, OUCP showed its significant effects as compared to MCP and SXCP in significant manner (*p* < 0.01 and *p* < 0.05, respectively).

#### Biochemical Estimation of Antioxidant Parameters

**Figure 10:**
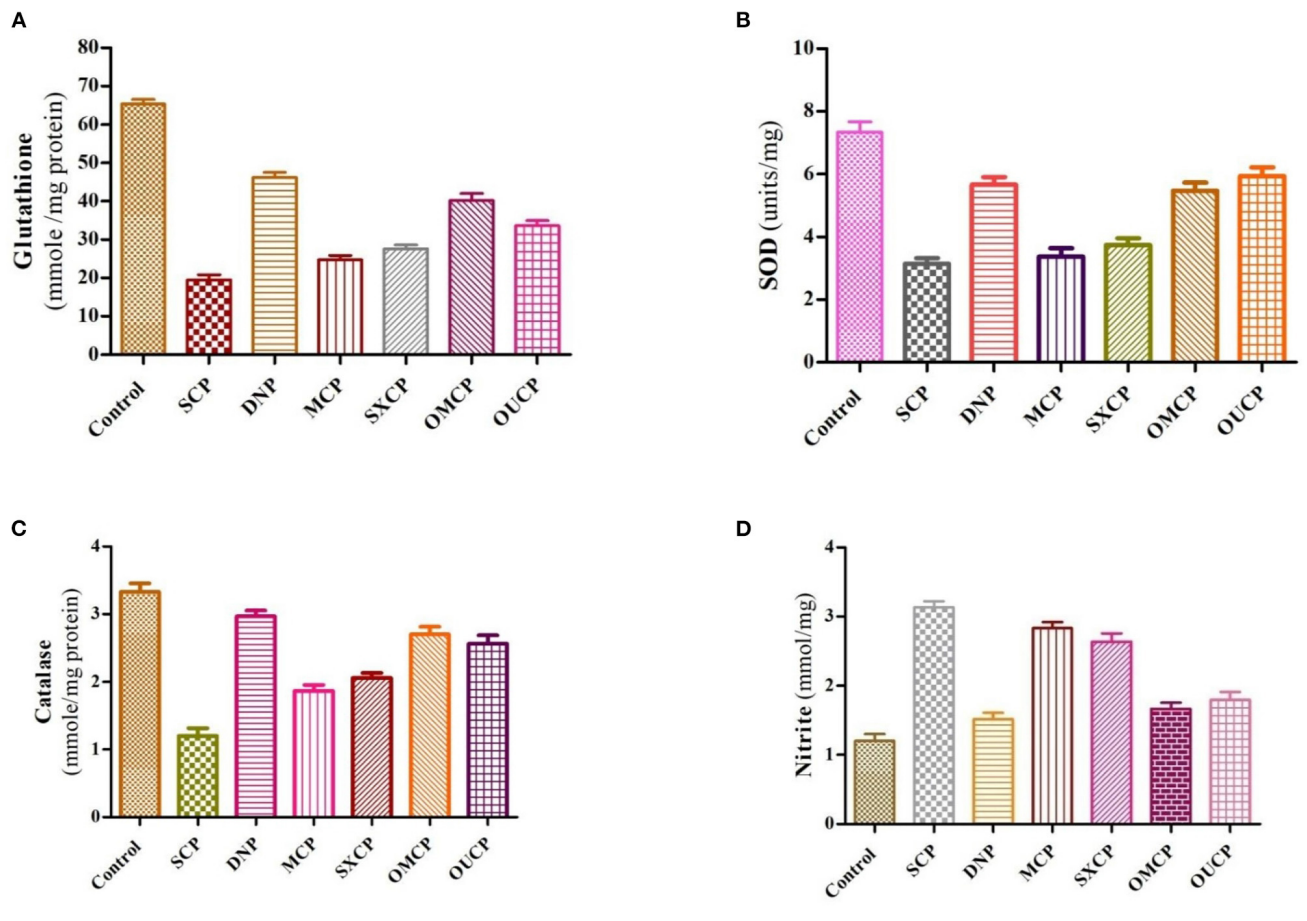
Biochemical estimation of different anti-oxidant stress parameters **(A)** reduced glutathione **(B)** SOD **(C)** catalase **(D)** nitrite content in various groups.

The free radicals generated are affiliated with excess oxidative stress which was observed in the pathogenesis of Alzheimer's diseases leading to aging along with cell apoptosis (69). Several research studies in past proposed the involvement of reactive oxygen species (ROS), in development of Alzheimer's disease (70–72). Hence, in this study, the effects of optimized extracts of CP seeds on biochemical parameters such as reduced glutathione, SOD, catalase, and nitrite content were evaluated (Figure 10). Scopolamine significantly impaired learning and memory and increased oxidative stress level in animals. Moreover, scopolamine significantly reduces the concentration of glutathione, SOD, and catalase level as compared to control group and increased the nitrite content (*p* < 0.01). The OMCP significantly increased the reduced glutathione level as compared to CEM (*p* < 0.01). OUCP also significantly increased the glutathione (*p* < 0.05) as compared to conventional methods (maceration). Further, OMCP increases the catalase activity as compared to MCP and SXCP (*p* < 0.01 and *p* < 0.05, respectively). The catalase concentration was also significantly enhanced by OUCP as compared to MCP and SXCP (*p* < 0.05, *p* < 0.01, respectively). OMCP also alters the SOD significantly as compared to CEMs, MCP, and SXCP (*p* < 0.01 and *p* < 0.05, respectively). Meanwhile, OUCP also significantly increased the SOD concentration (*p* < 0.01) as compared to CEMs. The concentration of nitrite content by OUCP and OMCP was reduced in a significant manner (*p* < 0.01) as compared to extracts obtained by CEM. Furthermore, results indicated that CP extracts obtained at optimized conditions changed the oxidative stress level in a significant manner as compared to CEM (*p* < 0.01 and *p* < 0.05), respectively. In the literature, evidences are present which indicated that alteration in behavioral and biochemical parameters occurs due to the stressful living conditions (73, 74). Our observations also supported the behavioral recovery and positive change in the oxidative stress levels, concomitant with the previous published reports (75, 76). The phytoconstituents present in *CP* seeds are mostly fatty acid esters, phenolic compounds, sesquiterpenes, and flavonoids and are responsible for various pharmacological activities such as antioxidants and memory enhancing activities (7, 9, 77, 78). Esters such as palmitic acid present in the different extracts also possess the antioxidant potential and effectively protect the fatty tissue from peroxidation in brain (79). Also, the past reports claimed that polyphenols neutralize free radicals in brain by crossing BBB to protect nervous system and improve the cognitive functions (80). Moreover, a positive correlation has been established in past between the total phenolic content and free radical scavenging potential of extracts (81, 82). Hence, the improved protective effect against oxidative stress could be attributed to enhanced concentration of different plant actives including the TPC and TFC in OMCP and OUCP.

#### AChE Inhibitory Potential of Different Extracts

The deterioration of cholinergic neurons majorly present in the basal forebrain was observed to be affiliated with the depletion of acetylcholine neurotransmitter (83, 84). The patients of Alzheimer's disease suffer from a censorious element to produce dementia due to reduced level of acetylcholine (85). The recent advancement focuses on the modulation of AChE activity. Various AChE inhibitor drugs have been reported for symptomatic treatment of Alzheimer's diseases, namely, physostigmine, donepezil, heptyl physostigmine, tacrine, and galantamine (86). Several researchers have reported that these AChE inhibitors enhance the production of acetylcholine at the cholinergic synapses, lowering the symptoms of dementia and stimulation of cognitive activity in human as well as animals (87–89).

**Figure 11:**
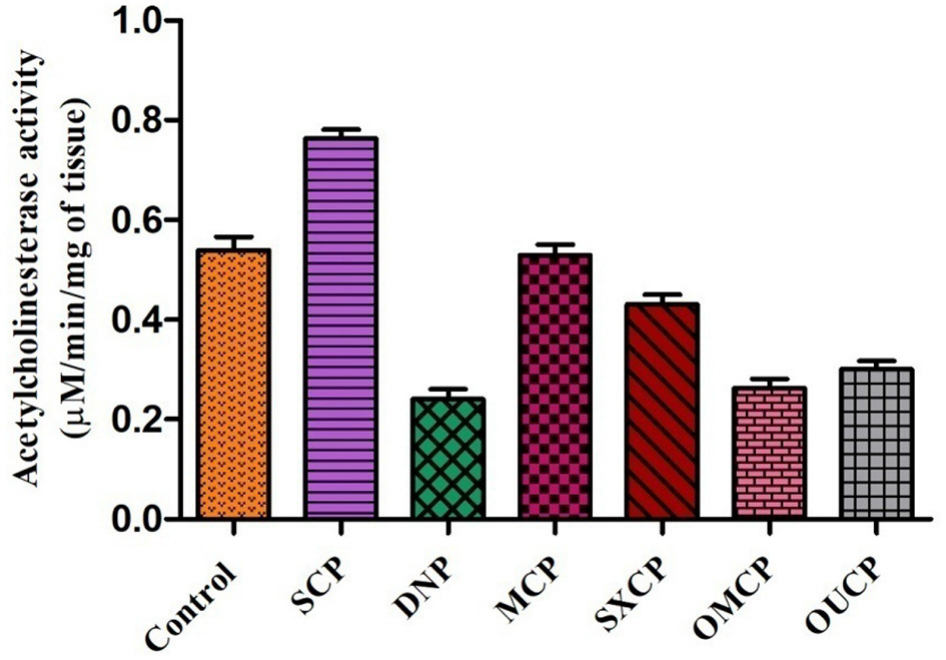
AChE inhibitory potential of different extracts and standard drugs.

The administration of different extracts for 14 consecutive days produced a significant decrease in AChE activity as compared to control. AChE concentration in brain tissue of animals of different groups was measured and represented as bar diagram (Figure 11). As compared to control group, scopolamine significantly increased AChE concentration as compared to control (*p* < 0.01). OMCP and OUCP significantly reduced the AChE concentration as compared to scopolamine group (*p* < 0.01). Moreover, the AChE inhibition also significantly reduced by the optimized extracts as compared to extracts obtained in CEM (*p* < 0.05).

On interpretation of data, we can say that optimized extracts of selected herb in green methods strongly exhibited the AChE inhibition potential as compared to conventional extracts. Previously, the selected plant was also reported to possess the anti-AChE potential but the improved action of the optimized extracts (OMCP and OUCP) could be attributed to enhanced concentration of plant actives including the TPC and TFC (90, 91). Moreover, numerous sesquiterpenes from natural sources are also reported to possess the AChE potential and play significant role in the prevention of Alzheimer's diseases (86). Also, the agonist of muscarinic receptors such as pilocarpine that exhibited the cholinomimetic property could play role in the Alzheimer's disease (92). GC-MS analysis indicated that along with other constituents, the concentration of phytocompounds such as different sesquiterpenes (agarofurans, celorvicol) and pilocarpine also improved significantly which could be responsible for the better memory enhancing the potential of green extracts.

## Conclusion

The demand for the herbal extracts has scaled up tremendously in the recent years. The selected medicinal plant, *CP* Willd (Jyotishmati), has enormous pharmacological potential. Also, the seed extract of the plant is largely used in the development of traditional ayurvedic formulations for the improvement of learning and memory. The herb is generally extracted by the conventional methods but to meet the rising demand of extract and looking at the limitation of conventional techniques, the improved and non-conventional methods are always researched upon. In this study, the green methods such as MAE and UAE were used to extract the plant. As in past, these techniques were not only proved to be the environmental friendly but also reported to improve the EY. Here also, the extraction at the optimized conditions (microwave power 268.91 watts, time 6.83 min, solvent concentration 80%, and solvent to solid ratio of 41.43 ml in MAE and sonication time of 11.81 min, solvent concentration 90%, and volume 27.36 ml in UAE) intensified the extract yield. Moreover, GC-MS analysis led to the improvement in concentration of different fatty acid esters, alkaloid, sesquiterpenes, and steroidal compounds. Further, the optimized extracts also positively altered the behavioral recovery, stress parameters, and AChE concentration, and the results are concomitant with the previous published reports. Also, the extraction process in green methods completed in significantly less time as compared to conventional methods. In nutshell, it can be concluded that selected green methods could be possibly scaled up with necessary modifications at the commercial level to meet the global demand of extract.

## Data Availability Statement

The original contributions presented in the study are included in the article/supplementary material, further inquiries can be directed to the corresponding authors.

## Ethics Statement

The research protocol was approved by Institutional Animal Ethical Committee (IAEC), MDU, Rohtak, vide reference number 1767/RE/S/14/CPCSEA/CAH/76-85 dated 26-02-2021.

## Author Contributions

AA: investigation, data curation, and original draft preparation. DK: analysis and writing—review editing. RA and SGB: writing—review editing. AS: software and writing—review editing. MA-D: conceptualization and writing—review editing. SB: software and writing—review editing. VM: conceptualization, supervision, and writing—review editing. All authors contributed to the article and approved the submitted version.

## Conflict of Interest

The authors declare that the research was conducted in the absence of any commercial or financial relationships that could be construed as a potential conflict of interest.

## Publisher's Note

All claims expressed in this article are solely those of the authors and do not necessarily represent those of their affiliated organizations, or those of the publisher, the editors and the reviewers. Any product that may be evaluated in this article, or claim that may be made by its manufacturer, is not guaranteed or endorsed by the publisher.
